# Disseminated sporotrichosis with osteoarticular involvement in a patient with acquired immunodeficiency syndrome: a case report

**DOI:** 10.1590/0037-8682-0120-2024

**Published:** 2024-12-16

**Authors:** Ana Paula Freitas Bahia dos Santos, Ana Carolina de Oliveira Mota, Gabriel Ramalho de Jesus, Matheus Dias Girão Rocha, Daniela de Freitas Pereira Calheiros Ângelo Durço, Luis Guilherme Rosifini Alves Rezende, Anna Christina Tojal da Silva, Fernando Crivelenti Vilar, Valdes Roberto Bollela, Roberto Martinez

**Affiliations:** 1 Faculdade de Medicina de Ribeirão Preto, Hospital das Clínicas, Departamento de Clínica Médica, Infectologia, Ribeirão Preto, SP, Brasil.; 2 Faculdade de Medicina de Ribeirão Preto, Hospital das Clínicas, Departamento de Ortopedia, Ribeirão Preto, SP, Brasil.; 3 Faculdade de Medicina de Ribeirão Preto, Hospital das Clínicas, Departamento de Patologia, Ribeirão Preto, SP, Brasil.

**Keywords:** Sporotrichosis, Acquired immune deficiency syndrome, Opportunistic infection

## Abstract

Sporotrichosis is caused by fungi belonging to the genus *Sporothrix*, and is generally acquired by traumatic inoculation. A 26-year-old man developed pustular lesions and a 6-kg weight loss after developing a lesion on his right hand 6 months previously. He was diagnosed with acquired human immunodeficiency syndrome (AIDS) and disseminated sporotrichosis cultures of bone and muscle biopsy and blood samples grew *Sporothrix schenckii*. The patient underwent reconstructive surgery and 9 months of treatment with amphotericin B and itraconazole, and showed complete wound healing and improved hand functionality. Suspicion of the disease is necessary in immunosuppressed patients living in endemic areas.

## INTRODUCTION

Sporotrichosis is a disease caused by fungi of the genus *Sporothrix*, which are found mainly in tropical and subtropical areas^1^ ^,^ ^2^, with the main agents being *S. schenckii*, *S. globosa* and *S. brasiliensis* ^2^ ^,^ ^3^. Infection generally occurs through traumatic inoculation of soil, plant, or other organic matter contaminated with the fungus; scratching by some mammals, such as cats and dogs^2^; or less commonly by inhalation of the microorganism^1^ ^,^ ^3^.

After inoculation, the fungus may remain in the subcutaneous tissue (fixed cutaneous or mucosal form), extend along adjacent lymphatic vessels (lymphocutaneous form), or spread hematogenously (disseminated cutaneous form). Extracutaneous forms are rare and difficult to diagnose and are associated with immunosuppression^1^ ^,^ ^4^ ^,^ ^5^.

In recent years, the prevalence of disseminated sporotrichosis has increased among people with human immunodeficiency virus (HIV) infection^2^. A literature search using the PubMed and SciELO databases confirmed that sporotrichosis with bone and joint involvement is uncommon and that the treatment and outcome are very variable. This case report highlights the need to suspect sporotrichosis in immunosuppressed patients living in endemic areas.

## CASE REPORT

**FIGURE 1:**
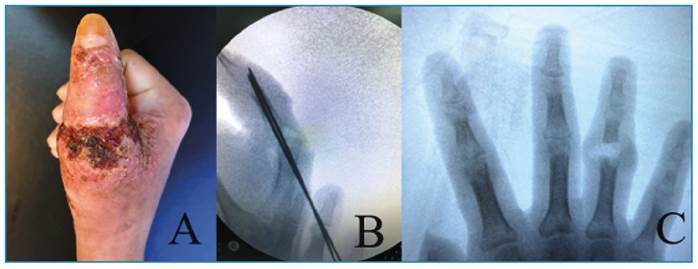
Appearance of the lesion to the right thumb at the time of the first treatment **(A)**. Thumb metacarpophalangeal arthrodesis **(B)**. Bone and proximal interphalangeal joint injuries of the fourth finger **(C)**.

A 26-year-old man from São José do Rio Preto, São Paulo, Brazil, had developed a contusion of the right hand after hitting it on a wooden door 6 months previously. The contusion subsequently became swollen and itchy, and pustular and crusted lesions developed on the skin (Figure 1A). Five months later, he developed similar circumscribed and crusted lesions on his back. He also experienced chronic rhinosinusitis and had a weight loss of 6 kg since the disease onset.

Another medical provider had diagnosed osteomyelitis and dorsal dislocation of the metacarpophalangeal joint of the right thumb and had performed debridement and arthrodesis. At that time, the patient was suspected to have HIV infection owing to the extent and chronicity of the lesions (Figure 1B). After the surgery, the patient was referred to a university hospital for investigation of the skin lesions and follow-up of the osteomyelitis. On admission, the patient was using ciprofloxacin, doxycycline, acyclovir, and fluconazole which had been prescribed for suspected bacterial osteomyelitis, herpes simplex, and cutaneous cryptococcosis.

On admission, blood tests revealed a hemoglobin level of 9.3 g/dL (reference value [RV]: 13.5-17.5 g/dL), hematocrit of 28% (RV: 39-50%), white blood cell count of 6,800 cells/µL (RV: 3,500-10,500 cells/µL), and platelet count of 317,000/µL (RV: 150,000-450,000/µL), with normal renal and hepatic function and electrolytes. The CD4+ T lymphocyte count was 65 cells/ µL and the HIV viral load (VL) was 98,860 copies/mL.

**FIGURE 2:**
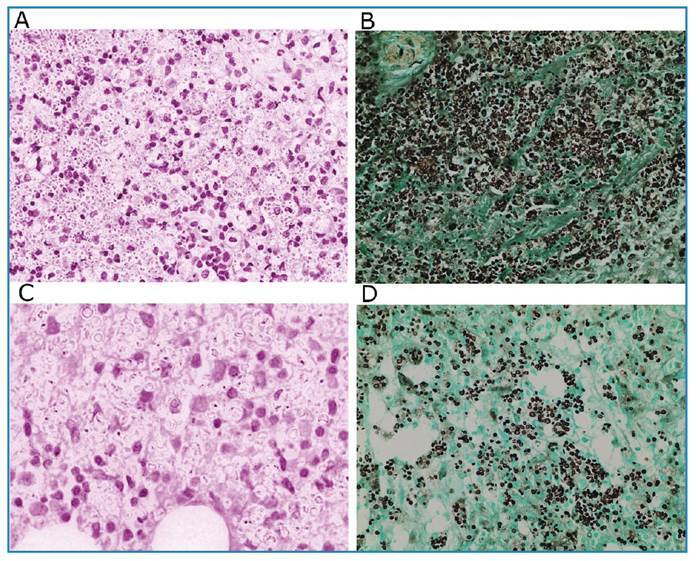
Histological section stained 400X with hematoxylin and eosin (HE) showing extensive infiltratration of the bone by macrophages containing a large number of oval intracytoplasmic structures with a clear eosinophilic outline permeated by lymphocytes and polymorphonuclear cells **(A)**. Silver impregnation with Gomori-Grocott stain (GMS) 400X in bone tissue showed intense infiltration by oval or globose unicellular yeasts containing one or more buds **(B)**. Histological section of connective tissue from the periarticular lesion stained with HE 1000X showing mononuclear inflammatory infiltrate and abundant oval structures **(C)**. GMS stained section 400X of the periarticular connective tissue lesion showing fungal structures in black **(D)**.

Three surgical approaches were required to debride the lesion, in addition to arthrodesis of the metacarpophalangeal joint of the thumb of the right hand and the proximal interphalangeal joint of the fourth finger of the right hand (Figure 1B and C). Histological aspects of the bone and soft tissue of the first finger of the right hand revealed numerous fungal structures in the cytoplasm of macrophages that were organized into granulomas (Figure 2). A culture of the same tissue revealed growth of *Sporothrix* sp., identified as *Sporothrix schenckii* using matrix-assisted laser-desorption ionization time-of-flight (MALDI-TOF) mass spectrometry. Furthermore, the same fungus was cultured from a biopsy specimen of the lesion on the back, and a nasal swab specimen. Counterimmunoelectrophoresis (CIE) was negative for anti-*Sporothrix* antibodies.

Initially, we decided to suspend fluconazole and start itraconazole 200 mg every 12 hours because of the diagnosis of disseminated sporotrichosis. Antiretroviral therapy (ART) was initiated with lamivudine, tenofovir, and dolutegravir, and itraconazole was initiated 4 days later. The patient continued to experience daily fever spikes after 7 days of itraconazole treatment, and *Sporothrix* sp*.* was identified in a blood culture; therefore, the antifungal treatment was changed to liposomal amphotericin B (2 mg/kg/day).

The patient experienced significant clinical improvement and defervescence after receiving a cumulative dose of 3 g of liposomal amphotericin B. He was discharged on itraconazole 600 mg/day, which was maintained for another 9 months. After 9 months, the dose was reduced to 400 mg/day and maintained for another 2 months. After 12 months of antifungal therapy the lesions had healed completely, and antifungal therapy was suspended.

**FIGURE 3:**
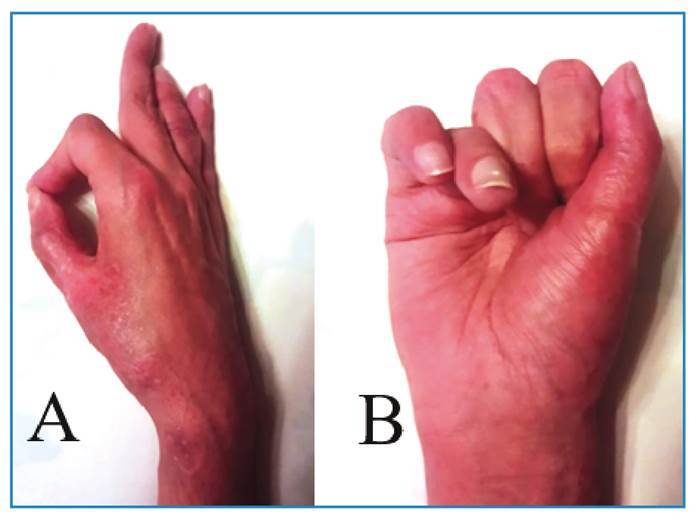
Hand function 6 months after surgery showing functional recovery of the ability to perform pinching **(A)** and palmar grip **(B)**.

The patient’s HIV VL became undetectable 12 weeks after starting ART, and his CD4+ count increased to 328 cells/µL after 10 months. The patient showed significant improvement in functionality of his right hand and was able to perform pinching and hand grip movements and return to performing activities of daily living (Figure 3).

## DISCUSSION

Impaired host immune responses are associated with increased severity of spirotrichosis^3^. In a review of 58 reported cases of HIV-associated sporotrichosis, Moreira *et al*.^2^ found a direct association between the CD4+ count and the clinical form of sporotrichosis, with all patients with disseminated disease having CD4+ counts below 200 cells/µL, as in our patient. In the same review, 56.9% of the cases were from Brazil, with a male predominance (84.5%)^2^.

In a review of 41 cases of bone disease in Brazil, 51% of the patients had a diagnosis of acquired human immunodeficiency syndrome (AIDS)^4^. *Sporothrix* was identified in 9 cases, which were all identified as *S. brasiliensis* ^4^, whereas in this case, the infection was caused by *S. schenckii*. In a case from the United States, published in 2018, a 33-year-old man with a history of alcoholism presented with sporotrichal arthritis of the knee^5^. As in our case, he underwent surgical debridement and was treated with itraconazole, resulting in complete healing. Although the organism did not undergo molecular identification, it was identified as *Sporothrix shenckii senso latu* ^5^. In a review published in 2014, 222 cases of bone and joint infections caused by dimorphic fungi were analyzed^6^. *Sporothrix schenckii* was the most frequent pathogen identified (84/222, 37.8%). Although *S. schenckii* was the most prevalent fungus, only histology and culture were performed to identify the microorganisms; therefore, they were probably from the genus *Sporothrix* and may have included other *Sporothrix* species^6^.

In cases of sporotrichosis, growth of the fungus on culture is the gold standard for the diagnosis. Molecular methods such as polymerase chain reaction (PCR) or MALDI-TOF can be used on identification and differentiation of closely related *Sporothrix* species^5^ ^,^ ^7^. An analysis by Oliveira *et al.* ^8^, MALDI-TOF clearly distinguished *Sporothrix brasiliensis, S. globosa, S. mexicana, S. schenckii, S. luriei* and *S. pallida*, enabling identification of all isolates at the species level^8^. Besides culture and molecular tests, serological tests based on the presence of antigens and antibodies (enzyme-linked immunosorbent assay [ELISA], CIE, or immunodiffusion) can also be used for diagnosis^7^ ^,^ ^9^. In our case, culture of biopsy tissue and blood, histopathology, and MALDI-TOF were essential for confirming the diagnosis. In our case, MALDI-TOF identified *S. schenckii* with good precision. A negative CIE for sporotrichosis may reflect the patient’s profound immunosuppression at the time.

The treatment of sporotrichosis varies according to its clinical form and the host’s level of immunosuppression. In the lymphocutaneous form, the drug of choice is oral itraconazole 200 to 400 mg/day for 2 to 4 weeks after the resolution of all lesions. Terbinafine 500 mg every 12 hours is an option for itraconazole failure^10^. In disseminated disease, the therapy of choice is amphotericin B, particularly the liposomal (3/mg/kg/day) and lipid complex (5 mg/kg/day) formulations, which have fewer side effects than the deoxycholate formulation^11^. Itraconazole at a dose of 200 mg every 12 hours for 12 months is recommended as sequential therapy in cases of a good response; however, immunosuppressed patients may require lifelong therapy. Itraconazole 200 mg every 12 hours for at least 12 months is the first option for the osteoarticular form, whereas lipid amphotericin B with a subsequent transition to itraconazole is recommended in cases of extensive disease or non-response to initial therapy^10^. In our case, initial therapy was performed with liposomal amphotericin B, followed by itraconazole for a further 12 months, and a clinical cure was achieved.

The ideal time to start ART in patients diagnosis with HIV and sporotrichosis has not been established; however, the start of ART can be delayed in patients at high risk of immune reconstitution syndrome (central nervous system disease, low CD4+ count, and high HIV VL)^2^.

In conclusion, we present a case of disseminated *S. schenckii* infection affecting the bloodstream, skin, and musculoskeletal system in a patient with advanced HIV infection. The diagnosis was challenging owing to other possible diagnoses, including deep mycoses, tuberculosis, and non-tuberculous mycobacteria. Clinicians should be aware of sporotrichosis as a possible opportunistic infection in patients with advanced HIV disease, especially in those from endemic regions or with an epidemiological.
